# Hydrocortisone Reduces 28-day Mortality in Septic Patients: A Systemic Review and Meta-analysis

**DOI:** 10.7759/cureus.4914

**Published:** 2019-06-17

**Authors:** Waqas J Siddiqui, Praneet Iyer, Ghulam Aftab, FNU Zafrullah, Muhammad A Zain, Kadambari Jethwani, Rabia Mazhar, Usman Abdulsalam, Abbas Raza, Muhammad O Hanif, Esha Sharma, Sandeep Aggarwal

**Affiliations:** 1 Cardiology / Nephrology, Drexel University College of Medicine, Philadelphia, USA; 2 Internal Medicine, University of Tennessee Health Sciences Center, Memphis, USA; 3 Internal Medicine, Orange Park Medical Center, Orange Park, USA; 4 Internal Medicine, Steward Carney Hospital, Tufts University School of Medicine, Boston, USA; 5 Internal Medicine, Sheikh Zayed Medical College and Hospital, Rahim Yar Khan, PAK; 6 Internal Medicine, Drexel University, Philadelphia, USA; 7 Internal Medicine, Steward Carney Hospital, Boston, USA; 8 Nephrology, Drexel University, Philadelphia, USA; 9 Internal Medicine, George Washington University, Washington D.C., USA

**Keywords:** hydrocortisone, sepsis, septic shock, fludrocortisone, mortality, meta-analysis

## Abstract

The goal of this study was to determine the utility of hydrocortisone in septic shock and its effect on mortality. We performed a systematic search from inception until March 01, 2018, according to PRISMA (Preferred Reporting Items for Systematic Reviews and Meta-Analyses) guidelines comparing hydrocortisone to placebo in septic shock patients and selected studies according to our pre-defined inclusion and exclusion criteria. Four reviewers extracted data into the predefined tables in the Microsoft Excel (Microsoft Corp., New Mexico, US) sheet. We used RevMan software to perform a meta-analysis and draw Forest plots. We used a random effects model to estimate risk ratios. A two-sided p-value of ≤ 0.05 was considered statistically significant. A total of five randomized control trials (RCTs) with 5,838 patients were included in our analysis. The primary outcome was mortality at 28 days. Secondary outcomes were intensive care unit (ICU) and in-hospital mortality, mortality at 90 days and one year, reversal of shock, intensive care unit (ICU) and hospital length of stay, incidence of superinfections, and incidence of limb and/or cerebral ischemia. The 28-day mortality was significantly reduced with hydrocortisone, 808 vs. 880 with placebo, Risk Ratio (RR)=0.92, confidence interval (CI) =0.85-0.99, p=0.04, I^2^=0%. There was no difference in ICU mortality (RR=0.93, CI=0.81-1.08), in-hospital mortality (RR=0.95, CI=0.84-1.08), 90-day mortality (RR=0.93, CI=0.84-1.02, p=0.10), and one-year mortality (RR=0.97, CI=0.84-1.12). Superinfections were significantly common with hydrocortisone, RR=1.16, CI=1.05-1.28, p=0.003. In conclusion, the use of hydrocortisone showed a significant reduction in mortality at 28 days and a trend toward reduced ICU mortality. This mortality reduction was observed at the cost of significantly higher superinfections.

## Introduction

Sepsis is a significant health concern globally with an associated mortality of 14.7% to 29.9% [1]. Over the years, although the number of sepsis cases and total mortality has increased, the associated case fatality rate has decreased [1].^ ^Other than the respiratory support with mechanical ventilation, hemodynamic support with fluid resuscitation and vasopressors, and source control of the infection with antibiotics and surgical evacuation of infection, there is no additional approved treatment for either sepsis or septic shock [2].^ ^Steroids have been evaluated as a treatment option for several years. The suggested theory behind the use of steroids is that they suppress inflammatory mediators and treat sepsis-induced relative adrenal insufficiency, which has been studied in various trials and is yet to be proved [3-4].

A study from 1976 by Schumer et al. showed a significant reduction in mortality in septic shock with high dose steroids given for a short duration [5].^ ^However, several subsequent studies were unable to replicate these findings but, in turn, showed increased associated mortality due to a higher incidence of superinfection, defined as a new clinical infection that occurred during therapy or within ten days of discontinuation of antimicrobial agents [6-8]. The use of steroids was discouraged until 2002 when the French study by Annane et al. showed significant mortality benefit with the use of steroids, which brought steroids back in favor [9]. Several subsequent studies, including systematic reviews, meta-analysis, and randomized control trials (RCTs), have not shown consistent evidence for or against steroids in sepsis and septic shock [10-12].^ ^Current surviving sepsis guidelines recommend the use of steroids when fluid resuscitation and vasopressors are not effective in correcting hemodynamic instability, but this remains a weak recommendation [2]. The two recent RCTs evaluating the use of hydrocortisone in septic shock patients suggested conflicting results leaving clinicians with no explicit guidance [13-14].^ ^There have been no meta-analysis to date which only utilized RCTs for systematic review and meta-analysis. The earlier systematic reviews have used studies, which had a variable patient population with systemic inflammatory response syndrome, sepsis, or septic shock. Those meta-analysis included RCTs, non-randomized trials, observational retrospective, and prospective studies and studies from the 1980s and 1990s where they also used dexamethasone, prednisone, and methylprednisone instead of only potent mineral corticoids, which are hydrocortisone and fludrocortisone. They also included studies in which steroids were used for reasons other than septic shock (e.g., meningitis). Our meta-analysis only incorporates RCTs, which included patients only with septic shock and utilized hydrocortisone alone or with fludrocortisone. Recently published RCTs in The New England Journal of Medicine (NEJM) on March 1, 2018, have not been part of any of the prior systematic reviews and meta-analyses, and the editorial published in the same edition of NEJM recommended to decide about patient management on the basis of subsequent meta-analyses utilizing these two RCTs [15].

## Materials and methods

We conducted this meta-analysis is to identify the effect of potent mineralocorticoids (hydrocortisone and fludrocortisone) in refractory septic shock patients with possible underlying relative adrenal and mineral corticoid insufficiency and their impact on short-term (defined as 28-day) mortality. The other steroids lack significant mineral corticoid activity as compared to hydrocortisone and fludrocortisone. For the primary outcome, we also performed the sub-group analysis by time to administration of hydrocortisone from time to randomization (early, i.e., within eight hours of randomization vs. late, i.e., within 24-72 hours of randomization). Our secondary outcomes assessed the long-term survival with the intensive care unit (ICU) and the hospital mortality and length of stay (LOS) with reversal of shock. We also evaluated the difference in commonly encountered complications of septic shock, including the incidence of superinfections and limb and cerebral ischemic events.

We completed a systematic review according to the PRISMA (Preferred Reporting Items for Systematic Review and Meta-Analyses) guidelines [16]. We searched the MEDLINE and PubMed databases from inception until March 01, 2018, only for RCTs, comparing the use of hydrocortisone to the placebo in septic shock patients.

Our search strategy included (glucocorticoid OR hydrocortisone OR steroid) AND (sepsis OR septic OR septic shock). We used the Boolean operator ‘OR’ to combine the search terms.

Inclusion criteria

1) Prospective RCTs, 2) Comparing hydrocortisone with or without fludrocortisone to placebos in patients with documented septic shock, 3) Patients age ≥ 18 years, 4) At least 100 patients were randomized in the study, and 5) At least one endpoint was 28-day mortality.

Exclusion criteria

We excluded non-randomized and retrospective studies, studies which used steroids other than hydrocortisone, and total number of patients was <100; studies that included patients with sepsis and severe sepsis, were in the non-English language, lacked 28-day mortality data, and in which both arms received hydrocortisone.

Primary endpoints

The primary endpoint was mortality at 28 days.

Secondary endpoints

We analyzed the following secondary endpoints: 1) Mortality in ICU; 2) Mortality in the hospital; 3) Mortality at 90 days; 4) Mortality at one year; 5) Reversal of Shock; 6) ICU LOS; 7) Hospital LOS; 8) Incidence of limb and/or cerebral ischemia; and 9) Incidence of superinfection.

Data extraction and quality assessment

**Figure 1:**
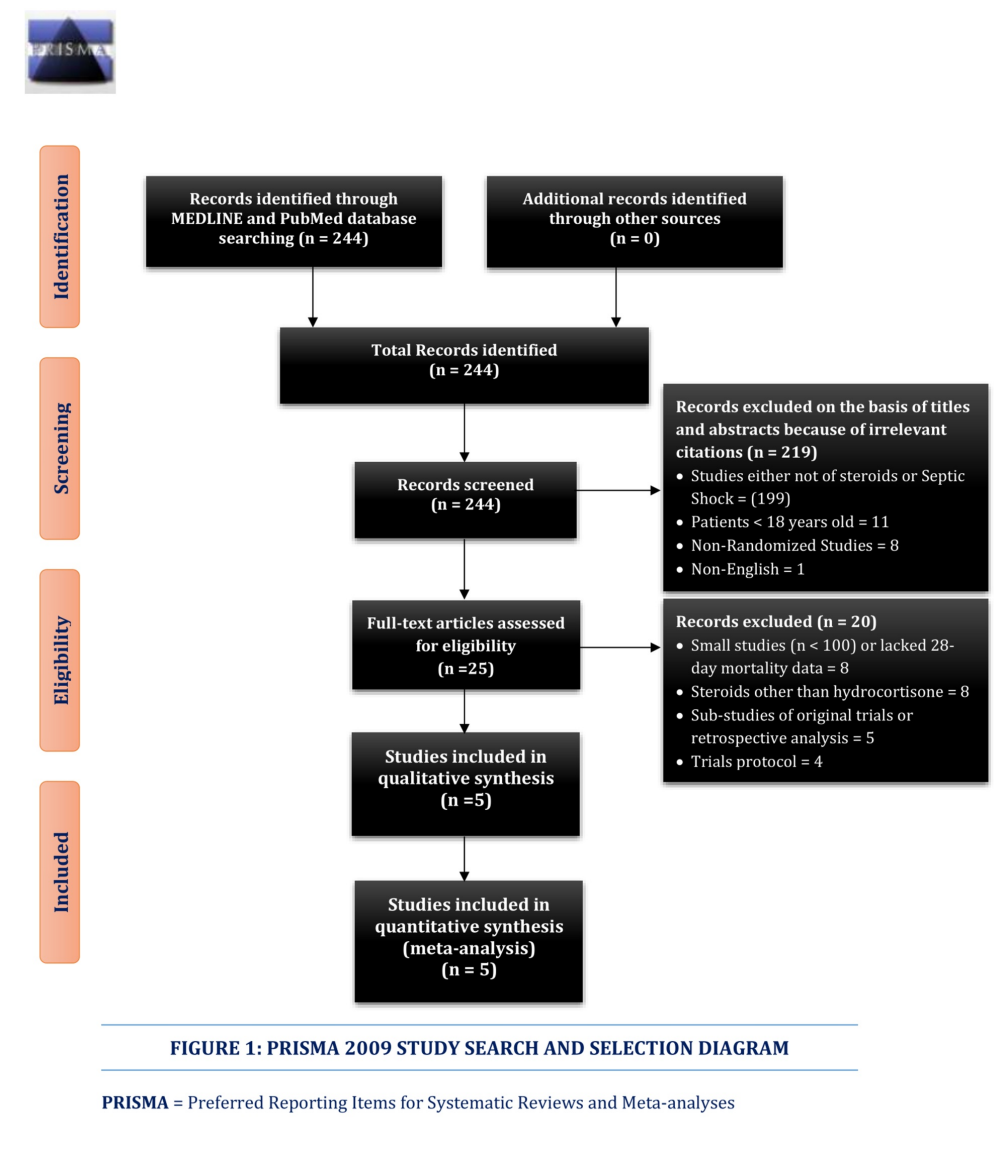
PRISMA 2009 Study Search and Selection Diagram PRISMA: Preferred Reporting Items for Systemic Reviews and Meta-analyses

**Figure 2:**
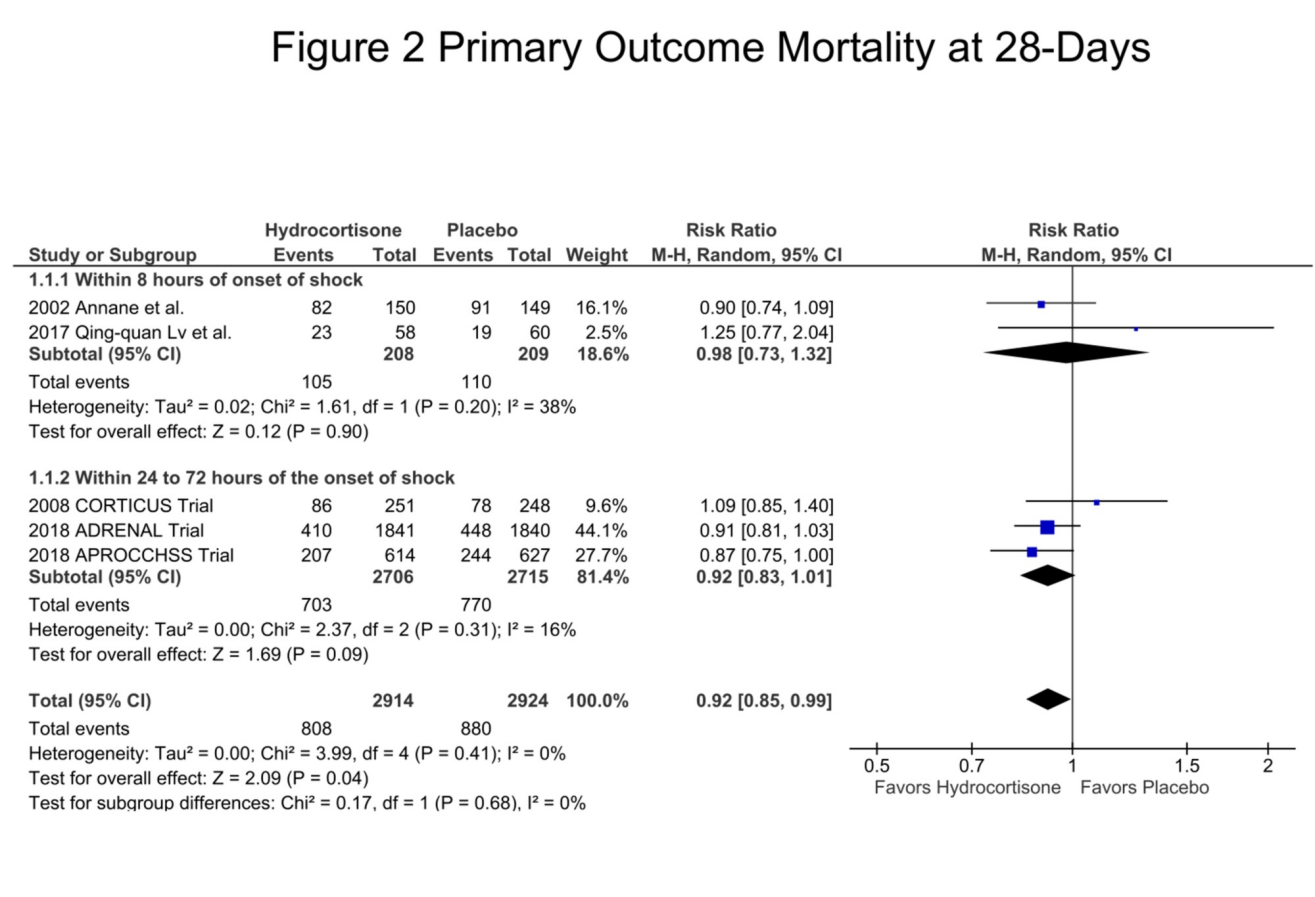
Primary Outcome - Mortality at 28 Days

Four reviewers, W.J.S., A.R., U.A.S., and M.O.H. extracted the data in the predefined data fields in the Excel sheet for baseline characteristics and study outcomes. They added outcomes that were mentioned in the outcomes tables and described in the text. W.J.S. cross-checked all the entered data and made corrections where necessary. All four reviewers agreed with the corrections and the final entry. Table 1 shows the features and differences of individual RCTs, and Table 2 summarizes the baseline characteristics of individual trials [9,11-14,17]. We used Cochrane collaboration’s tool risk assessment of bias in randomized trials for the quality assessment of RCTs [18] (Figures 1-2 and Table 3).

**Table 1:** Outcomes

Outcome	Effect Estimate	Confidence Interval	p-value	I^2 ^(%)
Primary Outcome				
Mortality at 28 days	0.92	0.85 – 0.99	0.04	0
Secondary Outcomes				
Mortality in Intensive Care Unit After sensitivity analysis	0.93 0.87	0.81 - 1.08 0.78 – 0.97	0.35 0.01	52 0
Mortality in the hospital	0.95	0.84 – 1.08	0.41	39
Mortality at 90 days	0.93	0.84 – 1.02	0.13	37
Mortality at one year	0.97	0.84 – 1.12	0.67	46
Reversal of Shock	1.17	0.74 – 1.86	0.5	24
Intensive Care Unit Length of Stay	0.89	-2.56 to 4.33	0.61	0
Hospital Length of Stay	1.58	-4.23 to 7.38	0.59	0
The incidence of Superinfection	1.15	1.04 – 1.27	0.008	0
The incidence of limb and/or cerebral ischemia	1.32	0.30 – 5.90	0.72	0

**Table 2:** Characteristics of Randomized Control Trials RCT = Randomized Control Trial, F/u = Follow up, n. = number, NEJM = New England Journal of Medicine AJEM: American Journal of Emergency Medicine. JAMA: Journal of the American Medical Association, NZ: New Zealand, KSA: Kingdom of Saudi Arabia, UK: United Kingdom; ICU: Intensive Care Unit, IV = intravenous, w/o = without

Name	Design	Country	Publication Year	Journal	Enrollment	Population	Time to randomization from the onset of shock	Setting	Intervention Vs. Comparison	Dose and Type of Steroid and Route of Administration	F/u Duration	Average 28-day Mortality across studies n. /total (%)			
2018 APROCCHSS trial [14]	Double-blind placebo-controlled RCT	France	3/1/18	NEJM	September 2008- June 2015	Septic shock	Within 24 hours of onset of shock	ICU	Hydrocortisone plus Fludrocortisone vs. Placebo	50 mg IV Q6 hours plus 50 µg 9-α-fludrocortisone via NG tube for 7 days w/o tapering	180 days	451/1271 (35.8)	
2018 ADRENAL trial [13]	Double-blind placebo-controlled RCT	UK, NZ, KSA, Australia, Denmark	1/19/18	NEJM	March 2013- April 2017	Septic shock	Within 24 hours of onset of shock	ICU	Hydrocortisone vs. Placebo	200 mg/d as a continuous IV Infusion for 7 days	90 days	858/3681 (23.3)	
2017 Qing-quan Lv et al. [11]	Double-blind placebo-controlled RCT	China	6/4/17	AJEM	September 2015 - September 2016	Septic shock	Within 6 hours of onset of shock	ICU	Hydrocortisone vs. Placebo	200 mg/d as a continuous IV Infusion for 6 days, then tapered during a 6-day period	28 days	41/120 (34.2)	
2008 CORTICUS trial [17]	Double-blind placebo-controlled RCT	Austria, Israel, Belgium, UK, Germany, France, Portugal, Netherlands	1/10/08	NEJM	March 2002-November 2005	Septic shock	Within 72 hours of onset of shock	ICU	Hydrocortisone vs. Placebo	50 mg IV Q6 hours for 5 days; then tapered during a 6-day period	28 days	164/499 (32.9)	
2002 Annane et al. [9]	Double-blind placebo-controlled RCT	France	8/21/02	JAMA	September 1995 - March 1999	Septic shock	Within 8 hours of onset of shock	ICU	Hydrocortisone plus Fludrocortisone vs. Placebo	50 mg IV Q6 hours plus 50 µg 9-α-fludrocortisone via NG tube for 7 days w/o tapering	28 days	173/299 (57.8)	

**Table 3:** Baseline Characteristics of Individuals Trials no. = Number, SD = Standard Deviation; SAPS = Simplified Acute Physiology Score; SOFA = Sequential Organ Failure Assessment; APACHE = Acute Physiology and Chronic Health Evaluation; SIRS = Systemic Inflammatory Response Syndrome; Pao2:Fio2 = the ratio of the partial pressure of arterial oxygen to the fraction of inspired oxygen; COPD = Chronic Obstructive Pulmonary Disease; CAD = Coronary Artery Disease; DM = Diabetes Mellitus; CKD = Chronic Kidney Disease; ICU = Intensive Care Unit; IV = Intravenous; N/A = Data not available

Studies	2018 APROCCHSS trial [14]	2018 ADRENAL trial [13]	2017 Qing-quan Lv et al. [11]	2008 CORTICUS trial [17]	2002 Annane et al. [9]					
Treatment arms	Hydrocortisone + Fludrocortisone	Placebo	Hydrocortisone	Placebo	Hydrocortisone	Placebo	Hydrocortisone	Placebo	Hydrocortisone + Fludrocortisone	Placebo
N	614	627	1853	1860	58	60	251	248	150	149
Male sex — no. (%)	402 (65.5)	424 (67.7)	1119 (60.4)	1140 (61.3)	33 (56.9)	37 (61.7)	166 (66)	166(67)	96(64)	104(70)
Age — years Mean ± SD	66±14	66±15	62.3±14.9	62.7±15.2	68.8±12.6	64.8±16.7	63±14	63±15	62(15)	60(17)
Whites - no. (%)	N/A	N/A	N/A	N/A	N/A	N/A	236 (94)	228 (92)	137 (92)	139 (95)
Admissions from Medical Ward no. (%)	495 (82.4)	499 (81)	1273 (68.8)	1266 (68.2)	17 (41.5)	22 (57.9)	80 (32)	93 (38)	89(59)	90(60)
Admissions from Surgery No. (%)	N/A	N/A	576 (31.2)	591 (31.8)	N/A	N/A	169 (67.8)	153 (62)	61(40.7)	59(39.6)
SAPS II	56±19	56±19	N/A	N/A	N/A	N/A	49.5±17.8	48.6±16.7	60(19)	57(19)
SAPS III	N/A	N/A	N/A	N/A	N/A	N/A	N/A	N/A	N/A	N/A
APACHE II Score Mean ± SD	N/A	N/A	24	23	25.5±9.5	21.3±6.9	N/A	N/A	N/A	N/A
SOFA Score Mean ± SD	12±3	11±3	N/A	N/A	11.9±3.3	9.9±3.0	10.6±3.4	10.6±3.2	N/A	N/A
SIRS Criteria, No. /Total no. (%)										
Temperature ≤36 ^o^ C or ≥ 38 ^o^ C	N/A	N/A	N/A	N/A	N/A	N/A	N/A	N/A	N/A	N/A
Temperature ^o^ C	N/A	N/A	N/A	N/A	N/A	N/A	37.9±1.5	38.0±1.4	38.0±2	37.9±2.2
Heart rate Mean ± SD or > 90 beats/min	N/A	N/A	96±21.6	95±20.9	N/A	N/A	119±26	118±25	118±21	118±21
Mean arterial pressure — mm Hg	N/A	N/A	72.5±8.2	72.2±8.3	N/A	N/A	N/A	N/A	54±10	55±10
Systolic Blood Pressure - mm Hg	N/A	N/A	N/A	N/A	N/A	N/A	94±23	95±27	N/A	N/A
Central venous pressure — mm Hg	N/A	N/A	12.0±5.2	12.1±5.3	N/A	N/A	N/A	N/A	N/A	N/A
Lowest mean arterial pressure — mm Hg	N/A	N/A	57.3±8.5	57.1±9.1	N/A	N/A	N/A	N/A	N/A	N/A
Highest lactate level — mg/dl	N/A	N/A	34.2±29.1	34.5±28.2	N/A	N/A	3.9±3.6	4.1±4.1	4.6±4.4	4.3±4.3
Highest bilirubin level — mg/dl	N/A	N/A	1.7±2.4	1.7±2.4	N/A	N/A	N/A	N/A	N/A	N/A
Highest creatinine level — mg/dl	N/A	N/A	2.2±2.0	2.1±1.7	N/A	N/A	N/A	N/A	N/A	N/A
Lowest Pao2:Fio2	N/A	N/A	164.6±91.3	166.4±91.9	N/A	N/A	162±89	154±73	176±120	171±124
Highest white-cell count — cells ×10−9/liter	N/A	N/A	17.4±11.4	17.8±14.7	N/A	N/A	N/A	N/A	N/A	N/A
Tachypnea, hypocapnia, Mechanical vent	N/A	N/A	N/A	N/A	N/A	N/A	N/A	N/A	N/A	N/A
Leukocytosis, leukopenia, left shift	N/A	N/A	N/A	N/A	N/A	N/A	14.9±9.8	14.7±9.8	13.1±10.1	13.0±8.4
Patients with comorbidities, no. (%)	N/A	N/A	N/A	N/A	54 (93.1)	49 (81.7)	N/A	N/A	N/A	N/A
Hypertension	N/A	N/A	N/A	N/A	25 (43.1)	26 (43.3)	89(35)	98(40)	44(29)	40(27)
COPD	N/A	N/A	N/A	N/A	2 (3.4)	4 (6.7)	27(11)	29(12)	17(11)	24(16)
CAD	N/A	N/A	N/A	N/A	7 (12.1)	8 (13.3)	37(15)	47(19)	20(13)	11(7)
DM	N/A	N/A	N/A	N/A	14 (24.1)	12 (20.0)	51(20)	56(23)	20(13)	17(11)
CKD	N/A	N/A	N/A	N/A	2 (3.4)	1 (1.7)	22(9)	21(9)		
Malignancy	N/A	N/A	N/A	N/A	9 (15.5)	13 (21.7)	47(19)	37(15)	23(15)	18(12)
Community Acquired Infection	468 (77.7)	459 (75.5)	N/A	N/A	N/A	N/A	N/A	N/A	94(63)	93(62)
Nosocomial, ICU	N/A	N/A	N/A	N/A	N/A	N/A	N/A	N/A	N/A	N/A
Nosocomial, Ward	N/A	N/A	N/A	N/A	N/A	N/A	N/A	N/A	N/A	N/A
Nosocomial	N/A	N/A	N/A	N/A	N/A	N/A	N/A	N/A	30(20)	34(23)
Site of Infection no. (%)										
Unknown	11 (1.8)	18 (2.9)	145 (7.9)	136 (7.3)	7 (12.1)	4 (6.1)	N/A	N/A	2(1)	0
Lung	373 (60.7)	363 (58)	623 (33.8)	677 (36.5)	22 (37.9)	22 (36.7)	N/A	N/A	61(41)	70(47)
Abdomen	74 (12.1)	68 (10.9)	477 (25.9)	467 (25.2)	21 (36.2)	34 (56.7)	N/A	N/A	26(17)	23(15)
Urinary Tract	102 (16.6)	118 (18.8)	146 (146 (7.9)	133 (7.2)	10 (17.2)	7 (11.7)	N/A	N/A	7(5)	7(5)
Skin and soft tissues	N/A	N/A	137 (7.4)	116 (6.3)	2 (3.4)	1 (1.7)	N/A	N/A	8(5)	12(8)
Bacteremia	N/A	N/A	N/A	N/A	18 (31.0)	13 (21.7)	N/A	N/A	39(26)	31(21)
Surgical wound	N/A	N/A	N/A	N/A	N/A	N/A	N/A	N/A	N/A	N/A
Positive blood culture no. (%)	225 (36.6)	229 (36.6)	316 (1.1)	325 (17.5)	42 (72.4)	44 (73.3)	N/A	N/A	39(26)	31(21)
Documented pathogen no. (%)	450 (73.3)	441 (70.4)	N/A	N/A	N/A	N/A	N/A	N/A	N/A	N/A
Gram-positive bacteria no. (%)	235 (38.3)	228 (36.4)	N/A	N/A	4 (6.9)	4 (6.7)	N/A	N/A	46 (31)	37 (25)
Gram-negative bacteria no. (%)	261 (42.5)	264 (42.2)	N/A	N/A	26 (44.9)	31 (51.7)	N/A	N/A	37 (25)	45 (30)
Adequate antimicrobial therapy no. (%)	595 (96.9)	595 (96.2)	1817 (98.3)	1821 (98.1)	48 (82.8)	47 (78.3)	N/A	N/A	137 (91)	141 (95)
Vasopressor administration										
Epinephrine										
No. of patients	53	58	134	113	N/A	N/A	35(14)	22(9)	41	31
Dose — μg/kg/min	2.31±6.62	1.74±2.41	N/A	N/A	N/A	N/A	0.6±1.2	0.9±2.6	0.8±0.7	1±0.9
Norepinephrine										
No. of patients	534	554	1823	1821	N/A	N/A	224(89)	231(93)	46	48
Dose — μg/kg/min	1.02±1.61	1.14±1.66	N/A	N/A	1.7±2.1	1.2±1.4	0.5±0.6	0.4±0.5	1.1±1.1	1.0±1.1
Glucocorticoids										
IV No./Total No. (%)	N/A	N/A	N/A	N/A	N/A	N/A	N/A	N/A	N/A	N/A
Hydrocortisone equivalent, (range), mg	N/A	N/A	N/A	N/A	N/A	N/A	N/A	N/A	N/A	N/A
Etomidate										
No. / Total no. (%)	N/A	N/A	N/A	N/A	N/A	N/A	22/251(8.6)	20/248(8.1)	N/A	N/A
Mean (SD), mg	N/A	N/A	N/A	N/A	N/A	N/A	N/A	N/A	N/A	N/A
Mechanical ventilation no. (%)	567 (92.3)	569 (91.3)	1845 (99.8)	1855 (99.9)	52 (89.7)	51 (85.0)	228(91)	212(86)	87(58)	75(50.3)
Renal-replacement therapy no. (%)	161 (27)	168 (28.1)	228 (12.3)	242 (13.0)	24 (41.4)	18 (30.0)	N/A	N/A	N/A	N/A
Organ failure n. (%)	N/A	N/A	N/A	N/A	10 (17.2)	6 (10.0)	N/A	N/A	N/A	N/A
Respiratory	N/A	N/A	N/A	N/A	7 (12.1)	4 (6.7)	N/A	N/A	N/A	N/A
Liver	N/A	N/A	N/A	N/A	1 (1.7)	1 (1.7)	N/A	N/A	N/A	N/A
Renal	N/A	N/A	N/A	N/A	3 (5.2)	1 (1.7)	N/A	N/A	N/A	N/A
Coagulation	N/A	N/A	N/A	N/A	3 (5.2)	1 (1.7)	N/A	N/A	N/A	N/A
Microcirculatory	N/A	N/A	N/A	N/A	N/A	N/A	N/A	N/A	N/A	N/A
Central nervous system	N/A	N/A	N/A	N/A	N/A	N/A	N/A	N/A	N/A	N/A

Data synthesis and analysis

Statistical Method 

We used a random effects model for our statistical analysis in RevMan Version 5.3 Copenhagen. We used the Mantel-Haenszel method for the statistical analysis of dichotomous data to calculate the risks ratio and inverse variance for the continuous data to estimate the mean difference. We reported our results using the effect estimate with 95% confidence interval. A two-sided p-value of ≤ 0.05 was considered statistically significant.

Heterogeneity

We used I2 and Chi2 statistics to estimate the heterogeneity with RevMan Version 5.3 Copenhagen. Variability between studies (inter-study) compared to variability within studies (intra-study) was assessed with the I2 statistic; I2 >50% indicates substantial heterogeneity as mentioned in the Cochrane Handbook for Systematic Reviews for Interventions, Version 5.1.0, Part 2: General Methods for Cochrane Reviews [19]. We performed a sensitivity analysis for substantial heterogeneity.

Study Selection

We identified 244 citations for RCTs. Two reviewers W.J.S. and P.I. reviewed the abstracts of each study and selected 25 articles and reviewed their full papers. They excluded 20 papers and selected five articles for qualitative and quantitative analysis comparing hydrocortisone to the placebo in patients with septic shock. Figure 1 shows the PRISMA study flow diagram and Table 4 summarizes the excluded studies failing to meet the inclusion criteria.

**Table 4:** Cochrane Risk of Bias for Quality Assessment

Name	Random Sequence	Allocation Concealment	Blinding of Participants and Personnel	Blinding of Outcome Assessment	Incomplete Outcome Data	Selective Reporting	
2018 APROCCHSS trial [14]	Yes Via Centralized Randomization Web site, stratified using permutation blocks Low Risk	Yes Low Risk	Yes Low Risk	Yes Low Risk	No Low Risk	No Low Risk
2018 ADRENAL trial [13]	Yes Password-protected, encrypted, Web-based Interface Low Risk	Yes Low Risk	Yes Low Risk	Yes Low Risk	No Low Risk	No Low Risk
2017 Qing-quan Lv et al. [11]	Yes Computer-generated random numbers Low Risk	Not Reported Unclear	Yes Low Risk	Not Reported Unclear	No Low Risk	No Low Risk
2008 CORTICUS trial [17]	Yes Computerized random-number generator Low Risk	Yes Low Risk	Yes Low Risk	Yes Low Risk	Yes High Risk	No Low Risk
2002 Annane et al. [9]	Yes Computer-generated random number Low Risk	Yes Low Risk	Yes Low Risk	Yes Low Risk	Yes One person withdrew consent after getting assigned treatment was excluded from analysis High Risk	No Low Risk

*Qualitative Analysis* 

We included five RCTs with 5,838 patients in our analysis. 2,914 patients were randomized to the hydrocortisone arm vs. 2,924 to the placebo arm. Two studies used Fludrocortisone in addition to hydrocortisone in the steroid arm [9,14]. (Table 5)

**Table 5:** Summary of Studies Excluded

Total Studies	Studies Included	Studies Excluded						
244	5	239						
Exclusion Criteria	Non-Randomized Studies	Steroids Other Than Hydrocortisone or Fludrocortisone	N of studies less than 100	Studies either not of steroids or Septic Shock	Non-English Language Studies	No reporting of Primary Outcome i.e. 28-day mortality	Studies which were Study Designs/Protocols	Age < 18 years
n.	8	8	4	199	1	4	4	11

## Results

Primary endpoints

See Table 1.

Mortality at 28 Days

There was a total of 1,688 deaths with a significantly reduced number of deaths in the hydrocortisone and fludrocortisone arm as compared to the placebo arm. There were 808 deaths in the hydrocortisone arm vs. 880 in the placebo arm, risk ratio (RR) = 0.92, confidence interval (CI) = 0.85 - 0.99, p = 0.04, I2 = 0 %, suggesting the mortality benefit at 28 days with hydrocortisone and fludrocortisone in septic shock patients. The sub-group analysis of the early administration of hydrocortisone, i.e., within eight hours of randomization showed no difference between the two groups, 105 in hydrocortisone group vs. 110 in the placebo arm, RR = 0.98, CI = 0.73 - 1.32, p = 0.90, I2 = 38%. The sub-group analysis of the late administration of hydrocortisone, i.e., within 24-72 hours of randomization showed a non-significant trend towards decreased mortality in the hydrocortisone arm, 703 vs. 770 in the placebo arm, RR = 0.92, CI = 0.83 - 1.01, p = 0.09, I2 = 16% (Figure 2).

Secondary endpoints

See Table 1.

Mortality in the ICU

**Figure 3:**
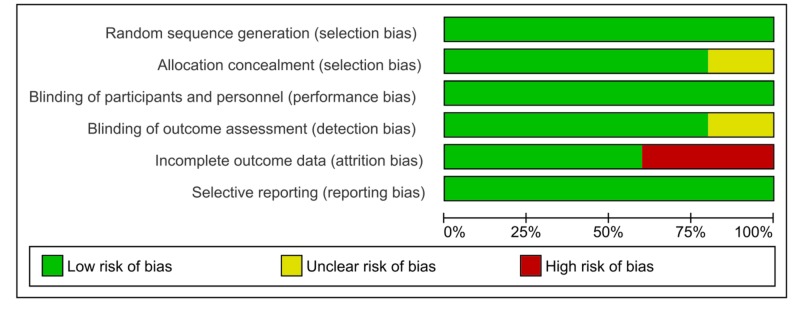
Risk of Bias Graph

There was a total of 856 deaths in the ICU with no difference in the number of deaths between the two groups, the hydrocortisone arm (409) vs. the placebo arm (447), RR = 0.93, CI = 0.81-1.08, p = 0.35, I2 = 52% (Figure 3). There was substantial heterogeneity between the two groups. On running the sensitivity analysis without the results of the Corticosteroid Therapy of Septic Shock (CORTICUS) trial, the results became statistically significant favoring hydrocortisone and fludrocortisone with I2 reducing to 0%. Hydrocortisone arm = 307 vs. placebo arm = 358 RR = 0.87, CI = 0.78-0.97, p = 0.01, I2 = 0%.

Mortality in the Hospital

**Figure 4:**
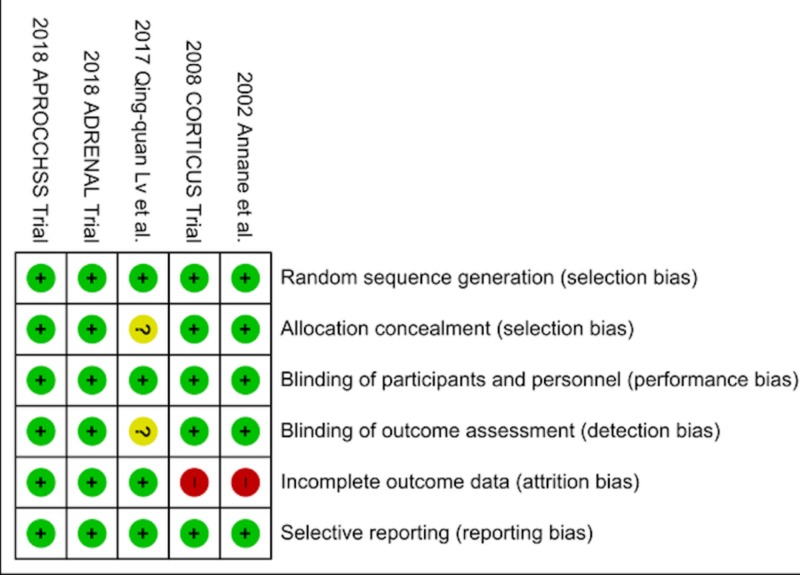
Showing Risk of Bias Summary

There were a total of 974 deaths during the hospital stay with no difference in either arm, 468 in the hydrocortisone arm vs. 506 in the placebo arm, RR = 0.95, CI = 0.84 - 1.08, p = 0.41, I2 = 39% (Figure 4).

Mortality at 90 Days

**Figure 5:**
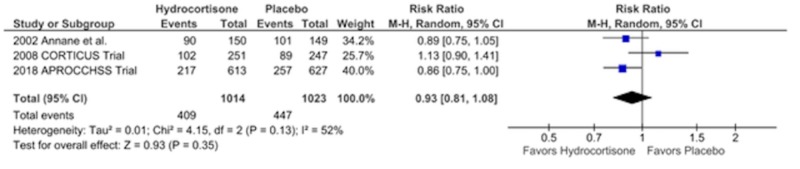
Forest Plot Showing Mortality in the Intensive Care Unit

Two trials reported 90-day mortality. There was a non-significant trend towards decreased mortality at 90 days in the hydrocortisone and fludrocortisone group, 775 vs. 834 in the placebo arm, RR = 0.93, CI = 0.84-1.02, p = 0.13, I2 = 37% (Figure 5).

Mortality at One Year

**Figure 6:**
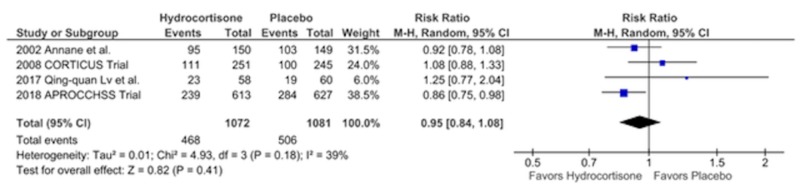
Forest Plot Showing Mortality in the Hospital

Two studies reported one-year mortality. There was no difference in mortality between the two groups at one year, 239 deaths in each arm, RR = 0.97, CI = 0.84-1.12, p = 0.67, I2 = 46% (Figure 6).

Reversal of Shock

**Figure 7:**
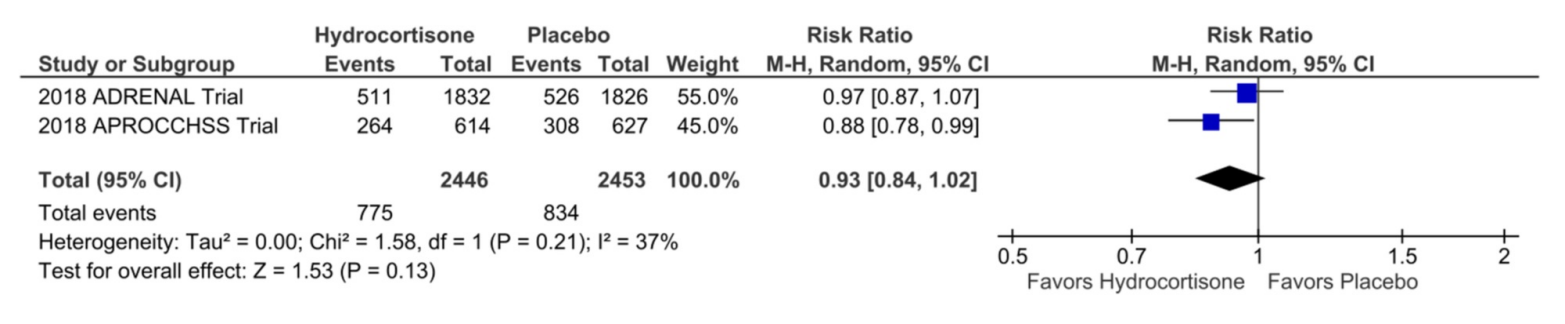
Forest Plot Showing Mortality at 90 Days

Two studies reported the shock reversal outcome. A total of 238 patients in the hydrocortisone group had shock reversal as compared to 226 in the placebo arm. There was no statistical difference between the two groups, OR = 1.17, CI = 0.74-1.86, p = 0.50, I2 = 24% (Figure 7).

ICU LOS

**Figure 8:**
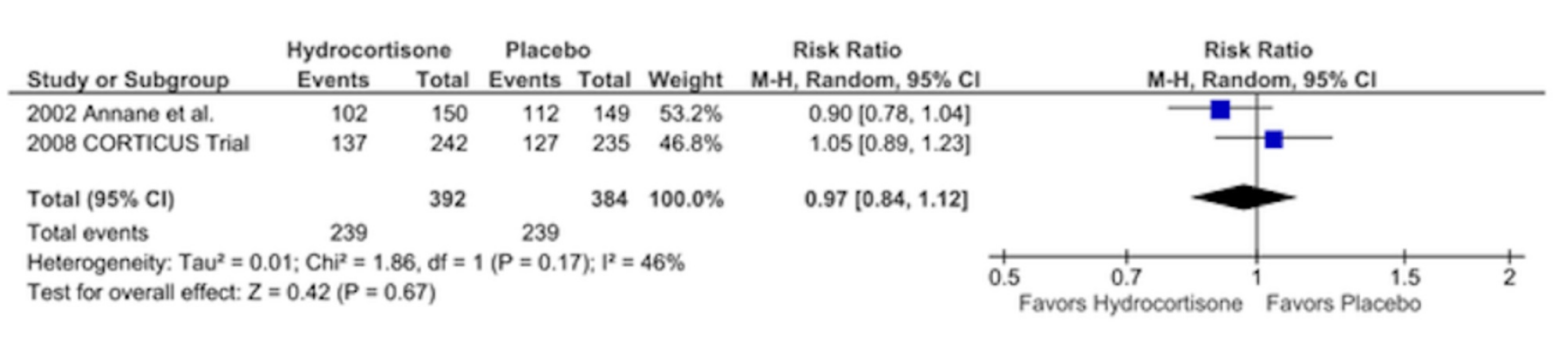
Forest Plot Showing Mortality at One Year

Only two studies reported the ICU LOS. There was no difference in the LOS in the ICU between the two groups, point estimate = 0.89 days, CI = -2.56 to 4.33, p = 0.61, I² = 0% (Figure 8).

Hospital LOS

**Figure 9:**
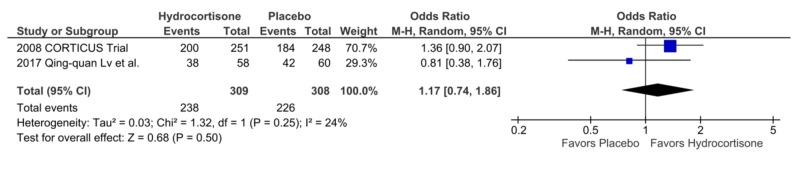
Forest Plot Showing Reversal of Shock

Two studies reported the LOS in the hospital. No statistical difference was observed in the two arms, point estimate = 1.58 days, CI = -4.23 to 7.38, p = 0.59, I² = 0% (Figure 9).

Incidence of Limb and/or Cerebral Ischemia

**Figure 10:**
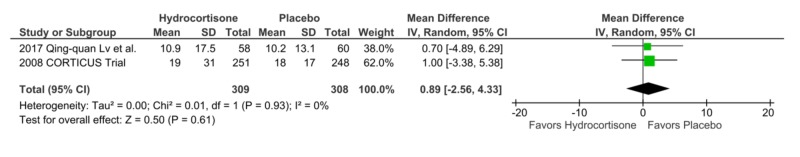
Forest Plot Showing the Length of Stay in the Intensive Care Unit

Two studies reported the incidence of either limb and/or cerebral ischemia. There was no difference in the two groups, four in the hydrocortisone arm as compared to three in the placebo arm, RR = 1.32, CI = 0.30-5.9, p = 0.72, I² = 0% (Figure 10).

Incidence of Superinfection

**Figure 11:**
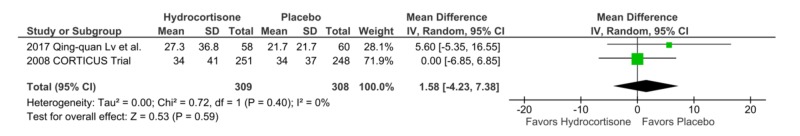
Forest Plot Showing the Length of Stay in the Hospital

**Figure 12:**
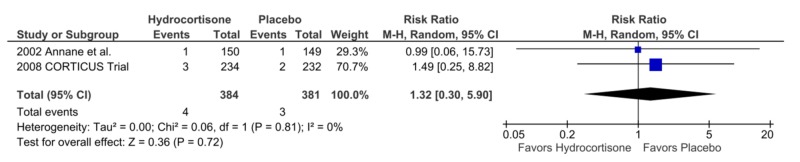
Forest Plot Showing the Incidence of Limb and/or Cerebral Ischemia

**Figure 13:**
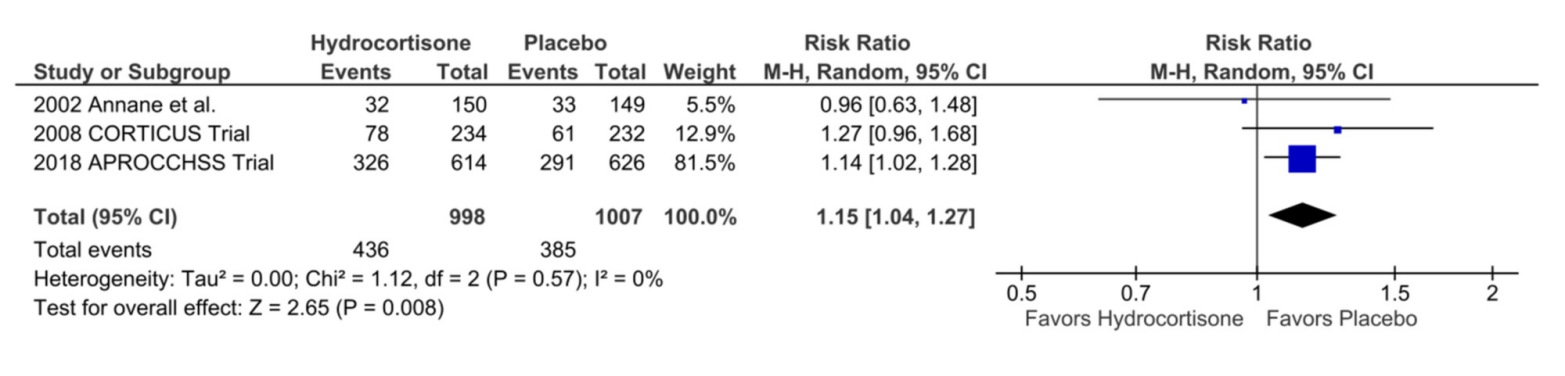
Forest Plot Showing the Incidence of Superinfections

Three studies reported the incidence of superinfection in the two treatment arms. Hydrocortisone was associated with a significantly higher number of superinfections as compared to placebo, 436 vs. 385, RR = 1.15, CI = 1.04-1.27, p = 0.008, I² = 0% (Figures 11-13).

## Discussion

The use of steroids in septic shock patients remains controversial due to the inconsistent results of previous trials and meta-analyses. Some authors believe that the significant variation in results could be due to dosing, duration of administration, and timing of initiation of steroids [20-21]. In pre-1997 trials, steroids were used in higher doses for a shorter duration. Subsequent studies were done after 1997 used steroids in physiological doses but for a longer duration, i.e., seven days. In the landmark study by Annane et al., steroids were started within three to eight hours after the diagnosis of septic shock, which led to a significant reduction in mortality [9]. However, in the CORTICUS trial, steroids were started within 12 hours of diagnosis, and no mortality benefit was observed [17]. Nonetheless, both of these trials showed an early reversal of shock in the steroid group. Due to the controversies surrounding the timing of steroid use noted in previous studies, Qing-Quan Lv et al. initiated steroids at the same time when vasopressors were started and found that reversal of shock was similar in both groups and there was no mortality difference either [11]. In the two recent landmark trials, ADRENAL (Adjunctive Corticosteroid Treatment in Critically Ill Patients with Septic Shock) and APROCCHSS (Activated Protein C and Corticosteroids for Human Septic Shock), steroids were started four and six hours after the initiation of vasopressors. Both trials also showed a reduction in pressor requirement with the use of steroids. Another point of debate has been regarding continuous infusion versus intermittent bolus dosing. The HYPRESS (Hydrocortisone for Prevention of Septic Shock) trial, which randomized patients with severe sepsis before developing septic shock, studied the continuous infusion of hydrocortisone with a taper over six days. It also failed to uncover any significant mortality benefit nor did it prevent the development of septic shock [12]. However, the surviving sepsis campaign guidelines recommend only intermittent bolus doses of hydrocortisone and not continuous infusion [2].

Amidst all this confusion, recently, two large multicenter RCTs were published to confirm or refute the findings of previous studies. In the ADRENAL trial, nearly 3,800 patients were randomized and assigned to receive a continuous infusion of either hydrocortisone or placebo. This study was adequately powered to determine a mortality difference [13]. In the APROCCHSS trial, a total of 1,241 patients were randomized to receive either a hydrocortisone - fludrocortisone combination or placebo [14]. The primary outcome in both trials was mortality at 90 days. The ADRENAL trial showed no significant mortality benefit at 90 days; on the other hand, in the APROCCHSS trial, a mortality benefit was noted in the hydrocortisone and fludrocortisone group. Both trials did show an early reversal of shock and rapid cessation of mechanical ventilation, which was similar to the results of earlier studies [9,17].

A review of these trials provides some additional insights into the subsets of patients who might benefit from the addition of corticosteroid therapy on top of conventional treatment for sepsis. The RCT with lower overall mortality (HYPRESS) had no mortality benefit, likely due to less sick patients (severe sepsis vs. septic shock) [12], and the trials with the highest mortality (French, APROCHSS) likely with the sickest patients, showed a mortality benefit [9,14]. This suggests that the addition of steroids may be helpful in patients who are “sicker,” and, in this case, unresponsive to conventional therapy of fluids, vasopressors, and antibiotics. Additionally, in the ADRENAL trial, there was a mortality benefit in the sub-group that received steroids after the first six hours, before which the patient, otherwise responsive to conventional goal-directed therapy, would be “selected out,” leaving patients who may have an additional benefit from corticosteroid therapy. Similarly, our sub-group analysis of the primary outcome on the basis of time to administration of hydrocortisone from randomization suggested a strong trend towards reduced mortality in the late administration group as compared to the early administration group (within eight hours vs. within 24 to 72 hours). Since the trials in the late administration group may also have patients who received hydrocortisone within eight hours of randomization could have led to a non-significant trend towards decreased mortality. These observations would remain speculative in the absence of a randomized trial looking at these specific outcomes and warrant a randomized clinical trial looking at hydrocortisone use in patients who are unresponsive to early goal-directed therapy. Such a trial would indeed be challenging to design and implement, given logistic and ethical issues.

Our study noted a statistically significant mortality benefit of the primary outcome of mortality at 28 days in the hydrocortisone group as compared to placebo. Also, there was a non-significant reduction in ICU mortality in the hydrocortisone arm, which became statistically significant after sensitivity analysis. This finding also suggests that the use of hydrocortisone incurs a mortality benefit in the ICU setting in addition to 28 days. However, the rates of superinfection were noted to be higher in the hydrocortisone group as compared to the placebo, which is consistent with the results of individual trials and older studies. The strength of our analysis primarily lies in study selection. We included only RCTs, which included patients with a septic shock, which compared hydrocortisone to placebo, and studies with at least a hundred patients in the trial. This is in contrast to earlier meta-analyses, which also included non-RCTs, cohort and retrospective studies, and studies with small population sizes with different steroids, including methyl-prednisolone, dexamethasone, betamethasone, hydrocortisone, and prednisone [10,20].

The limitations in performing this meta-analysis are: we included trials ranging from 2002 up until now. The management of sepsis and septic shock has evolved since early 2000, and so have the surviving sepsis guidelines [2,22-25], which is evident by the reduction in mortality from 57.8% in the French study [9] to 23.3% to 35.8% in subsequent studies [11,13-14,17]. Two out of five trials included fludrocortisone in addition to hydrocortisone. The recommendations from the American College of Critical Care Medicine and the surviving sepsis campaign in 2008 have reported that hydrocortisone has enough mineralocorticoid effects, making the administration of fludrocortisone irrelevant. Thus, we believe that the addition of fludrocortisone could not have provided a significant benefit to influence the results of our study [22,25]. The last limitation was the way steroids were administered in individual RCTs, bolus vs. continuous infusion.

## Conclusions

Our analysis showed a significant reduction in mortality at 28 days and a non-significant trend in ICU mortality and mortality at 90 days in the hydrocortisone group. The rates of superinfection were noted to be significantly higher in the hydrocortisone group. We believe there is still controversy over hydrocortisone, and we don’t know which patients, if any, should receive the drug. In the future, large RCTs are required before any new recommendations can be made comparing hydrocortisone to placebo, hydrocortisone plus fludrocortisone to placebo, and hydrocortisone to hydrocortisone plus fludrocortisone. Another potential trial can be designed comparing the time from the development of septic shock to the administration of hydrocortisone.
